# Association of phosphatase and tensin homolog low and phosphatidylinositol 3-kinase catalytic subunit alpha gene mutations on outcome in human epidermal growth factor receptor 2-positive metastatic breast cancer patients treated with first-line lapatinib plus paclitaxel or paclitaxel alone

**DOI:** 10.1186/s13058-014-0405-y

**Published:** 2014-07-24

**Authors:** Binghe Xu, Zhongzhen Guan, Zhenzhou Shen, Zhongshen Tong, Zefei Jiang, Junlan Yang, Michelle DeSilvio, Mark Russo, Meggan Leigh, Catherine Ellis

**Affiliations:** 1 Cancer Hospital, Chinese Academy of Medical Sciences and Peking Union Medical College, 5 Dong Dan San Tiao, Beijing, 100005 China; 2 Sun Yat-Sen Medical University Cancer Center, 651 Dongfeng East Road, Guangzhou, 510060 China; 3 Shanghai Cancer Center, Fudan University, 270 Dongan Road, Shanghai, 200032 China; 4 Tianjin Medical University Cancer Institute and Hospital, Huan-Hu-Xi Road, Tianjin, 300060 China; 5 Hospital affiliated to Military Medical Science, 8 Dongda Street, Beijing, 100071 China; 6 Beijing 301 PLA Hospital, 28 Fuxing Road, Beijing, 100853 China; 7 GlaxoSmithKline Oncology, 1000 Black Rock Road, Collegeville, 19426 PA USA

## Abstract

**Introduction:**

Phosphatidylinositol 3-kinase (PI3K) pathway deregulation (that is PIK3CA mutations and/or phosphatase and tensin homolog (PTEN) loss) has been shown to enhance breast cancer cell survival and confer resistance to chemotherapeutic agents. We studied the prognostic and predictive value of PIK3CA mutations and PTEN low in patients receiving paclitaxel alone or in combination with lapatinib.

**Methods:**

Immunohistochemistry and mutation analyses were used to evaluate PTEN and PIK3CA, respectively. Kaplan-Meier analysis with log-rank tests, logistic regression and Cox models were used in analyses of these biomarkers with efficacy endpoints.

**Results:**

In the overall population, PIK3CA mutations were associated with poorer overall survival (OS) (hazard ratio (HR) = 1.87; 95% confidence interval (CI): 1.22, 2.88; *P* = 0.001). PTEN expression was not associated with OS (*P* = 0.474). In the PIK3CA wild-type subgroup, lapatinib plus paclitaxel reduced risk of progression compared with paclitaxel alone (HR = 0.44; 95% CI: 0.28, 0.69; *P* <0.0001); progression-free survival (PFS) was not significantly improved within the PIK3CA mutation subgroup (*P* = 0.179). In the PTEN low group, OS was improved with addition of lapatinib (*P* = 0.039). In both PTEN subgroups, addition of lapatinib was associated with improvements in PFS (*P* <0.050). PIK3CA and PTEN were not predictive of treatment based on interaction tests (*P* >0.05).

**Conclusions:**

PTEN was neither a significant prognostic nor predictive factor. PIK3CA mutations were an adverse prognostic factor for survival but not predictive for lapatinib benefit.

**Trial registration:**

ClinicalTrials.gov NCT00281658 (registered 23 January 2006)

**Electronic supplementary material:**

The online version of this article (doi:10.1186/s13058-014-0405-y) contains supplementary material, which is available to authorized users.

## Introduction

Deregulation of the phosphatidylinositol 3-kinase (PI3K) pathway is frequently observed in many human cancers, and as a consequence is implicated in cancer pathophysiology. In breast cancer, gain-of-function mutations in the PI3K catalytic subunit alpha gene (PIK3CA) and loss of phosphatase and tensin homolog (PTEN) function are common genomic events, either of which can result in an activated PI3K pathway phenotype and induction of oncogenic transformation, as demonstrated in preclinical models [1]–[5]. These aberrations have been shown to be associated with clinicopathological characteristics including histological subtype, tumor grade, hormone receptor status and human epidermal growth factor receptor 2 (HER2) expression [5]–[7]. Additionally, studies have reported PIK3CA and PTEN as prognostic indicators and effectors of response to chemotherapies, endocrine therapy and HER2-directed therapies, (that is, trastuzumab and lapatinib) [2],[5],[6],[8]–[14]. The clinical relevance of PI3K pathway deregulation should be evaluated in the context of breast cancer molecular subtypes as these subtypes differ in survival outcome, treatment response, and frequency of PIK3CA mutations and PTEN loss [6],[15]–[18].

In breast cancers driven by HER2 the frequency of PIK3CA mutations or PTEN loss has been described as up to 40% [19]. The functional consequences of breast cancers harboring alterations in both HER2 and the PI3K pathway are most likely to provide a selective advantage in cellular processes that include cell growth and survival. More importantly, elucidating the impact PIK3CA mutations and/or PTEN loss have on the efficacy of HER2-directed agents is essential to the advancement of personalized medicine and the development of rational targeted therapy combinations with the aim to improve patient outcome.

Lapatinib, an orally administered, small molecule, reversible inhibitor of the intracellular tyrosine kinase domain of HER2, is currently approved for the treatment of HER2-positive metastatic breast cancer in combination with chemotherapy or aromatase therapy in postmenopausal women with hormone receptor-positive disease [20]. The effects of PIK3CA mutations and/or PTEN loss on the efficacy of lapatinib have been evaluated in preclinical and clinical studies. Evidence from one study evaluating the efficacy of lapatinib and trastuzumab in 18 HER2-amplified cell lines indicated that PI3K pathway activation correlated with trastuzumab resistance yet did not confer resistance to lapatinib in all cell line models, and that lapatinib activity was independent of these molecular features [21]. This finding was confirmed in clinical studies in patients with HER2-positive breast cancer, whereby PIK3CA mutations and PTEN loss were not significantly associated with lapatinib efficacy [22]–[25]. In contrast, other studies have reported that PTEN loss of function and PIK3CA mutations adversely impact lapatinib efficacy [9],[13]. Although results from several studies suggest the efficacy of lapatinib is independent of PIK3CA and PTEN, overall, the findings across studies are inconsistent.

We conducted exploratory analyses on the prognostic and predictive value of PI3K pathway activation in a large cohort of patients with HER2-positive metastatic breast cancer who received paclitaxel in combination with lapatinib or placebo in the randomized, Phase III study, EGF104535 (NCT00281658). We focused the analysis on PTEN protein expression as a measure of PTEN loss of function (PTEN low) and on three mutation hotspot regions in the PIK3CA gene (E542K and E545K/D in exon 9, which encodes the helical domain; H1047R in exon 20, which encodes the kinase domain), as these mutations are well characterized as constitutively activating and oncogenic.

## Methods

### Patient population and study design

The eligibility criteria and study design for EGF104535 have been previously reported [26]. Briefly, 444 patients from Brazil, China, Hong Kong, Pakistan, Peru, Russia, Thailand, and Ukraine with HER2 gene-amplified metastatic breast cancer, and who had not received prior therapy for metastatic disease, were stratified by hormone receptor status (estrogen receptor (ER)-negative and progesterone receptor (PgR)-negative vs. ER-positive and /or PgR-positive) and metastatic disease status (nonvisceral vs. visceral) then randomly assigned in a 1:1 ratio to receive first-line treatment with paclitaxel (80 mg/m^2^ weekly for three weeks of a four-week cycle) plus lapatinib (1500 mg once daily, continuously) or paclitaxel (80 mg/m^2^ weekly for three weeks of a four-week cycle) plus placebo (once daily, continuously).

The study (EGF104535) was performed in accordance with the Declaration of Helsinki and approved by local ethics committees, details of which have been previously reported [26]. All patients provided written informed consent to take part in the study. Additional consent was provided by patients who took part in the tumor genetics analysis.

### Specimen characteristics

Baseline tissue from primary breast tumor or metastatic site was obtained in the form of formalin-fixed, paraffin-embedded (FFPE) material (slides or blocks).

### Assay methods

Immunohistochemistry (IHC) staining using a rabbit monoclonal antibody against PTEN (138G6; Cell Signaling Technology, Danvers, MA, USA) in an analytically validated assay was used in the assessment of PTEN protein expression on FFPE whole tissue sections. Single pathology review (that is, two pathologists participated in the study; each case was reviewed by one pathologist) and image analysis were performed, thereby producing an ordinal score and optical density score, respectively. Staining intensity was allocated a score of 0, 1+, 2+, or 3 + .

Two approaches to PTEN analysis were considered based on the presence of cytoplasmic staining in invasive tumor cells. The primary analysis considered tumors scored IHC 0/1+ as having PTEN low. The secondary analysis considered tumors scored IHC 0 as exhibiting an absence of PTEN expression whereas those scored IHC 1+, 2+, or 3+ were considered as exhibiting any PTEN expression. PTEN immunoreactivity in the surrounding stroma acted as the internal positive control, a method consistent with that previously reported [12].

PIK3CA mutation test kit (Qiagen/DxS, Germantown, MD, USA) was used to assess mutation status on genomic deoxyribonucleic acid (DNA) isolated from FFPE tumor tissue. The assay detects exon 9 mutations E542K and E545D/K, and the exon 20 mutation H1047R. The presence of any one of the four PIK3CA mutations in a tumor was designated as PIK3CA mutation, whereas the absence of these four mutations was designated as PIK3CA wild-type.

A tumor was defined as having PI3K pathway activation if there was PTEN low, or a PIK3CA mutation or both.

### Study objective

The primary objective of this analysis was to evaluate the predictive and prognostic value of PIK3CA mutations and/or PTEN low in HER2-positive patients receiving first-line treatment with paclitaxel alone or in combination with lapatinib. The effect of PTEN low, PIK3CA mutation and PI3K pathway activation (PTEN low and/or PIK3CA mutation) was assessed in relevant clinical endpoints (overall survival (OS), progression-free survival (PFS), overall response rate (ORR), and clinical benefit rate (CBR)).

### Statistical analyses

The efficacy analyses were conducted on the intent-to-treat (ITT) population which comprised all randomized patients with available biomarker data.

The effect of PTEN low, PTEN any expression, PIK3CA mutation, and PI3K pathway activation on OS and PFS was assessed using a Cox proportional hazard model including the marker as a covariate. Multivariate Cox models adjusting for two stratification factors (hormonal status (hormone receptor-negative vs. hormone receptor-positive) and metastatic sites of disease (visceral vs. nonvisceral)) were performed to assess the interaction between treatment and the biomarker. The Wald chi-square test was used to test for the interaction. Kaplan-Meier survival curves were generated by biomarker status and treatment group; a log-rank test was used in the comparison between treatment arms. Association between PTEN low, PTEN any expression, PIK3CA mutation, PI3K pathway activation, and ORR and CBR was assessed using logistic regression models for each separately. For ORR, patients were divided into responder (complete response (CR), partial response (PR)), and non-responder groups (progressive disease (PD) or stable disease (SD)). For CBR, patients were divided into clinical benefit (CR, PR, or SD ≥24 weeks) and nonclinical benefit (SD <24 weeks, PD) groups. Unknown responses were excluded as these were exploratory analyses.

All tests comparing PIK3CA and PTEN with efficacy endpoints were two-tailed and *P* ≤0.05 was considered significant. The *P* values from these comparisons were not adjusted for multiple comparisons.

## Results

Baseline patient and tumor characteristics of the ITT population for study EGF104535 are shown in Table 1. Of the total study population, 390 patients had tissue available for biomarker analyses and 274 of these patients provided written informed consent for optional tumor genetics (that is, PIK3CA analysis). Of the 390 patient cases, 338 were from the primary tumor site (86.7%), 37 from a metastatic site (9.5%), and the site was not specified in 15 patient cases (3.8%); in the population who gave consent for PIK3CA analysis, the overall percentages were consistent with the population with tissue evaluable for biomarker analyses (specimen site out of 274 patient cases, 270 were primary (87.6%), 26 were metastatic (9.5%), and 8 were not specified (2.9%)).

**Table 1:** **Baseline patient and tumor characteristics (ITT population)**

Characteristics	Lapatinib + paclitaxel (*n* = 222)	Placebo + paclitaxel (*n* = 222)
**Median age, years (range)**	50.0 (25-74)	50.5 (26-73)
**Race,** ***n*** **(%)**		
Asian	192 (86)	192 (86)
Other	30 (14)	30 (14)
**ECOG performance status,** ***n*** **(%)**		
0	103 (46)	113 (51)
1	119 (54)	109 (49)
**Central HER2 FISH +** ^**a**^**,** ***n*** **(%)**	219 (99)	218 (99)
**Hormone receptor-positive,** ***n*** **(%)**	111 (50)	113 (51)
**Any visceral disease,** ***n*** **(%)**	187 (84)	186 (84)
**Median time since first diagnosis, months**	25.7	23.6
**Prior anticancer therapies** ^**b**^ **,** ***n*** **(%)**		
Any therapy	171 (77)	182 (82)
Chemotherapy	160 (72)	173 (78)
Adjuvant	146 (66)	161 (73)
Hormonal therapy	53 (24)	44 (20)
Biologic	0	4 (2)
Radiotherapy	94 (42)	85 (38)

^a^Five cases were HER2 FISH-borderline: three from lapatinib + paclitaxel, two from placebo + paclitaxel; two additional cases from placebo + paclitaxel never FISH-tested. ^b^A total of 12% of the study population had Stage IV breast cancer at initial disease presentation (14% in lapatinib + paclitaxel; 11% in placebo + paclitaxel). ECOG, Eastern Cooperative Oncology Group; FISH, fluorescence *in situ* hybridization; HER2, human epidermal growth factor receptor 2; ITT, intent-to-treat.

**Figure 1:**
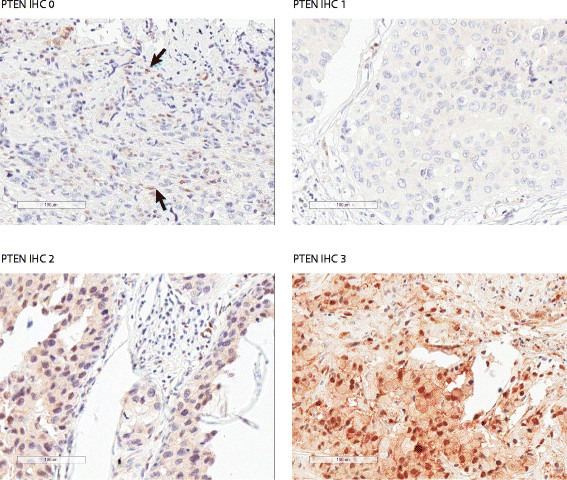
**Representative PTEN IHC staining.** Arrows point to stromal cells with positive PTEN staining. IHC, immunohistochemistry; PTEN, phosphatase and tensin homolog.

Evaluable results for PTEN and PIK3CA were available for 91% (355/390) and 77% (210/274) of patients with available tumor tissue and who provided consent for tumor genetics, respectively. In the cohort of patients with available tumor tissue and who provided consent, 24% (65/274) had tumors harboring PIK3CA mutations (E542K, E545K/D, and H1047R); 13% (49/390) of patients with available tumor tissue had absence of PTEN expression and 53% (205/390) had low PTEN expression. Results for PTEN expression and PIK3CA mutation analysis are summarized in Table 2. PTEN IHC staining representative of the four IHC scores are presented in Figure 1.

**Table 2:** **Summary of PTEN and PIK3CA results**

Characteristics	Lapatinib + paclitaxel (*n* = 222)	Placebo + paclitaxel (*n* = 222)
**Patients with available tumor tissue,** ***n*** **(%)**	198 (89)	192 (86)
**Tumors available for PTEN** ^**a**^ **,** ***n*** **(%)**	180	175
PTEN IHC 0	28 (16)	21 (12)
PTEN IHC 1+	76 (42)	80 (46)
PTEN IHC 0/1+	104 (58)	101 (58)
PTEN IHC 2+/3+	76 (42)	74 (42)
**Patients consented for PIK3CA** ^**b**^	141	133
**Tumors evaluable for PIK3CA** ^**a**^ **,** ***n*** **(%)**	104	106
PIK3CA mutation	29 (28)	36 (34)
PIK3CA wild-type	58 (56)	48 (45)
PIK3CA indeterminate	17 (16)	22 (21)
**Tumors with PTEN IHC 0/1+ and/or PIK3CA mutations** ^**c,d**^ **,** ***n*** **(%)**	119/180 (66)	117/175 (67)
**Tumors with PTEN IHC 0/1+ and PIK3CA mutation,** ***n*** **(%)**	14/104 (13)	20/106 (19)

^a^Nonevaluable findings were due to factors such as insufficient tumor tissue, absence of PTEN staining in stroma and poor DNA quality (PIK3CA). ^b^Somatic mutation analysis was optional. ^c^PIK3CA mutation frequency: 65% (42/65) were H1047R (35 cases are from primary tumor; 5 from metastatic; 2 from unknown site); 25% were E545K/D (14 cases are from primary tumor; 1 from metastatic, 1 from unknown site); 11% were E542K (7 from primary tumor). ^d^Defined as PI3K pathway activation. DNA, deoxyribonucleic acid; IHC, immunohistochemistry; PIK3CA, phosphatidylinositol 3-kinase subunit alpha gene; PTEN, phosphatase and tensin homolog.

### Effect of PIK3CA mutations and PTEN low on survival outcome

**Figure 2:**
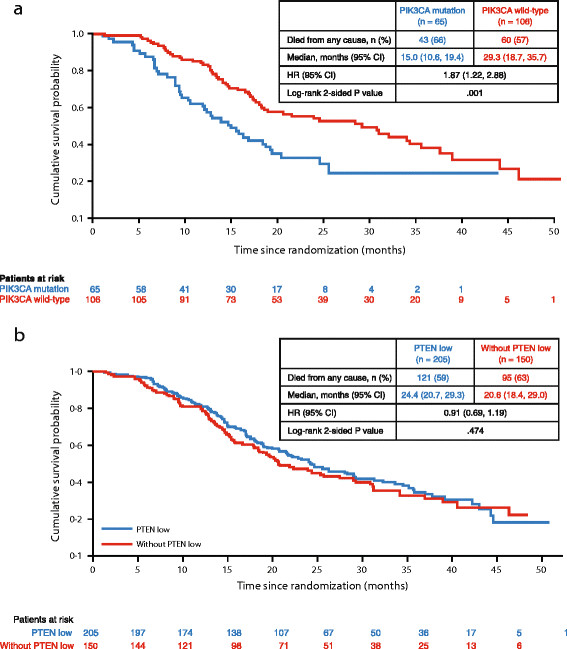
**OS outcome by: (a) PIK3CA mutation status; and (b) PTEN low.** OS, overall survival; PIK3CA, phosphatidylinositol 3-kinase subunit alpha gene; PTEN, phosphatase and tensin homolog.

In the overall study population, regardless of treatment, the presence of PIK3CA mutations was associated with significantly poorer OS (hazard ratio (HR) = 1.87; 95% confidence interval (CI): 1.22, 2.88; *P* = 0.001; Figure 2a), whereas PTEN expression was not associated with survival in this population (*P* = 0.474; Figure 2b). PI3K pathway activation, as defined by PTEN low and/or PIK3CA mutation, was not associated with OS (*P* = 0.085, data not shown).

PIK3CA mutations were significantly associated with poorer survival in the paclitaxel plus placebo population (*P* = 0.018, data not shown), whereas no statistically significant association between PIK3CA mutation and poorer survival was observed in the lapatinib plus paclitaxel population (*P* = 0.056, data not shown). PTEN low and PI3K pathway activation were not significantly associated with survival in either individual treatment arm (*P* >0.050, data not shown).

### Effect of PIK3CA mutations and PTEN low on lapatinib efficacy

**Figure 3:**
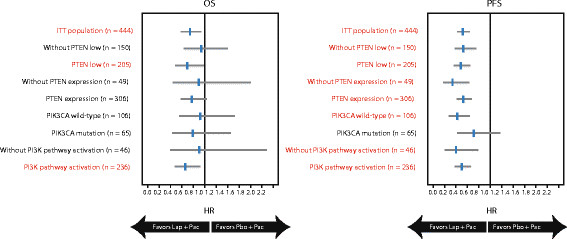
**Effect of PTEN and PIK3CA status on OS and PFS.** Groups in red text = statistical significance of *P* <0.050. HR, hazard ratio; ITT, intent-to-treat; OS, overall survival; PFS, progression-free survival; PI3K, phosphatidylinositol 3-kinase; PIK3CA, PI3K subunit alpha gene; PTEN, phosphatase and tensin homolog. PTEN low, IHC 1+/0; PTEN expression, IHC3+/2+/1+; PI3K pathway activation, PTEN low and/or PIK3CA mutation.

**Figure 4:**
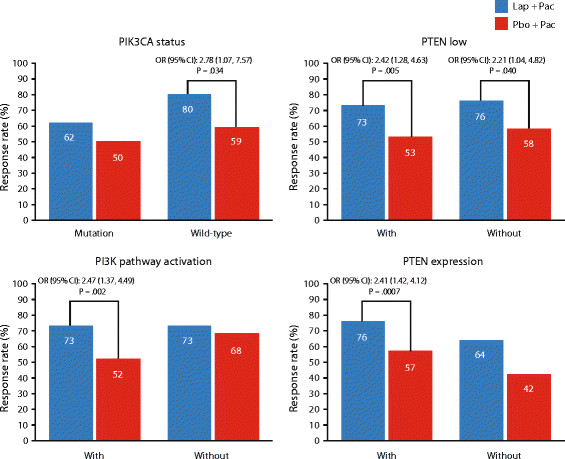
**Effect of PIK3CA and PTEN status on ORR.** CI, confidence interval; IHC immunohistochemistry; ORR, overall response rate; OR, odds ratio; PI3K, phosphatidylinositol 3-kinase; PIK3CA, PI3K subunit alpha gene; PTEN, phosphatase and tensin homolog; PTEN low, IHC 1+/0; PTEN expression, IHC3+/2+/1+; PI3K pathway activation, PTEN low and/or PIK3CA mutation.

**Figure 5:**
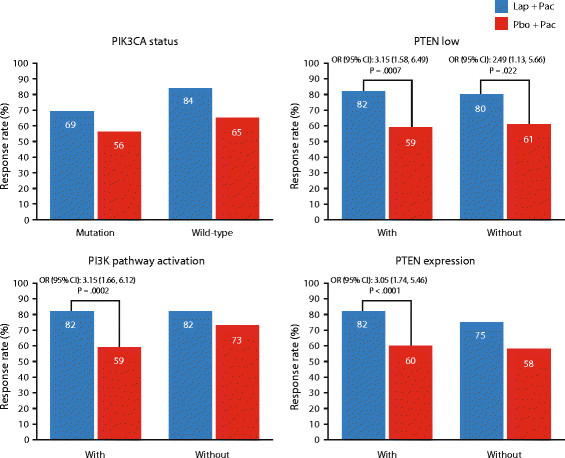
**Effect of PIK3CA and PTEN status on CBR.** CBR, clinical benefit rate; CI, confidence interval; IHC, immunohistochemistry; OR, odds ratio; PI3K, phosphatidylinositol 3-kinase; PIK3CA, PI3K subunit alpha gene; PTEN, phosphatase and tensin homolog; PTEN low, IHC 1+/0; PTEN expression, IHC3+/2+/1+; PI3K pathway activation, PTEN low and/or PIK3CA mutation.

In the PIK3CA wild-type subgroup, treatment with lapatinib plus paclitaxel reduced the risk of progression compared with paclitaxel alone (*n* = 106; HR = 0.44; 95% CI: 0.28, 0.69; *P* <0.0001); OS was not significant (*P* = 0.732) (Figure 3). Addition of lapatinib was also associated with significant improvement in ORR (*P* = 0.034) (Figure 4). CBR was numerically higher in patients treated with lapatinib-based therapy; however, the treatment difference was not significant (Figure 5).

In the PIK3CA mutation subgroup, a trend in a PFS benefit was shown in the lapatinib plus paclitaxel subgroup (HR = 0.71; 95% CI: 0.43, 1.17; *P* = 0.179); whereas, OS, ORR, and CBR were not significantly different between treatment groups (*P* >0.050) (Figures 3, 4 and 5).

OS was significantly improved in the PTEN low subgroup with addition of lapatinib (*P* = 0.039) (Figure 3), whereas OS was not significantly improved in the subgroup without PTEN low. Addition of lapatinib was associated with significant improvements in PFS, ORR, and CBR compared with paclitaxel alone in both PTEN subgroups (low/expression expression) (*P* <0.050) (Figures 3, 4 and 5).

PI3K pathway activation was associated with a significant improvement in PFS and OS with the addition of lapatinib to paclitaxel (*P* <0.0001; *P* = 0.013, respectively). In this same subgroup, significant improvements in ORR and CBR (*P* ≤0.002) were also observed in patients receiving lapatinib compared with paclitaxel alone (Figures 4 and 5).

### Predictive effect of PIK3CA mutations and PTEN low on lapatinib

In a multivariate analysis after adjusting for stratification factors, the interaction effect between PIK3CA and treatment in OS was not significant (*P* = 0.809); interaction effect in PFS was also not significant (*P* = 0.095). The interaction effect of PTEN low with treatment in OS and PFS was not significant (*P* = 0.088; *P* = 0.219, respectively) (Tables 3 and 4.)

**Table 3:** **Summary of Cox regression model for OS with treatment, PIK3CA mutation, PTEN low, and interaction adjusting for stratification factors**

Covariate	HR (95% CI)	Two-sided*P*value
**PIK3CA mutation** ^**a**^		
Treatment (Lap + Pac/Pbo + Pac) (*n* = 171)	0.88 (0.53, 1.46)	0.611
HR status (negative/positive)	0.82 (0.55, 1.23)	0.343
Metastatic sites (visceral/nonvisceral only)	0.52 (0.30, 0.92)	0.024
PIK3CA mutation (yes/no)	2.24 (1.29, 3.88)	0.004
Treatment^a^ PIK3CA interaction	0.91 (0.41, 2.02)	0.809
**PTEN low**		
Treatment (Lap + Pac/Pbo + Pac) (*n* = 355)	1.00 (0.67, 1.50)	0.998
HR status (negative/positive)	1.17 (0.89, 1.53)	0.256
Metastatic sites (visceral/nonvisceral only)	0.50 (0.33, 0.76)	0.001
PTEN low (yes/no)	1.16 (0.80, 1.69)	0.425
Treatment^a^ PTEN low interaction	0.62 (0.36, 1.07)	0.088

^a^Patients with a PIK3CA indeterminate tumor status were excluded. CI, confidence interval; HR, hazard ratio; Lap, lapatinib; OS, overall survival; Pac, paclitaxel; Pbo, placebo; PIK3CA, phosphatidylinositol 3-kinase subunit alpha gene; PTEN, phosphatase and tensin homolog.

**Table 4:** **Summary of Cox regression model for PFS with treatment, PIK3CA mutation, PTEN low, and interaction adjusting for stratification factors**

Covariate	HR (95% CI)	Two-sided*P*value
**PIK3CA mutation** ^**a**^		
Treatment (Lap + Pac/Pbo + Pac) (*n* = 171)	0.41 (0.27, 0.62)	<0.0001
HR status (negative/positive)	0.86 (0.62, 1.20)	0.379
Metastatic sites (visceral/nonvisceral only)	0.77 (0.50, 1.17)	0.218
PIK3CA mutation (yes/no)	1.31 (0.84, 2.07)	0.238
Treatment^a^ PIK3CA interaction	1.76 (0.91, 3.43)	0.095
**PTEN low**		
Treatment (Lap + Pac/Pbo + Pac) (*n* = 355)	0.59 (0.42, 0.83)	0.003
HR status (negative/positive)	1.01 (0.81, 1.26)	0.948
Metastatic sites (visceral/nonvisceral only)	0.66 (0.48, 0.89)	0.007
PTEN low (yes/no)	1.10 (0.81, 1.52)	0.537
Treatment^a^ PTEN low interaction	0.75 (0.48, 1.18)	0.219

^**a**^Patients with a PIK3CA indeterminate tumor status were excluded. CI, confidence interval; HR, hazard ratio; Lap, lapatinib; Pac, paclitaxel; Pbo, placebo; PIK3CA, phosphatidylinositol 3-kinase subunit alpha gene; PTEN, phosphatase and tensin homolog.

## Discussion

The effect of PIK3CA mutations and PTEN loss as prognostic factors has been evaluated extensively in breast cancer. However, in earlier studies these markers were evaluated in the absence of considering survival differences in breast cancer molecular subtypes. Additionally, the frequency at which these genetic alterations occur varies according to molecular subtype [19].

To the best of our knowledge, the present study is the largest of its kind evaluating the prognostic and predictive value of PIK3CA mutations and PTEN expression in patients with HER2-amplified metastatic breast cancer treated with lapatinib-based therapy. Overall, the prevalence of PIK3CA mutations and PTEN low was 31% and 58%, respectively, which is within the range of reported frequencies in HER2-positive breast cancer [14],[19],[27]–[31]. In the overall study population, and in patients in the paclitaxel plus placebo group, PIK3CA mutations were an adverse prognostic factor for survival, whereas in patients treated with paclitaxel plus lapatinib PIK3CA mutations were not significantly associated with OS outcome. In contrast, neither PTEN low alone nor when PIK3CA mutation and PTEN low were considered together were significantly associated with survival in any of the populations studied.

In a study that evaluated the association of PIK3CA status with clinicopathological factors in primary breast cancer, the effect of PIK3CA mutations was analyzed within molecular subtypes. In the overall population, as well as in the HER2-positive subtype, PIK3CA mutations conferred a metastasis-free survival benefit; however, in this study the HER2-positive population had not received a HER2-directed agent as part of their adjuvant treatment [6]. Whereas in a study of 240 patients with early-stage HER2-positive breast cancer treated with adjuvant trastuzumab-based therapy, PIK3CA mutation-positive breast cancer was associated with significantly poorer OS. Furthermore, no significant association was observed with low PTEN and OS or invasive disease-free survival (DFS) [29]. The lack of a significant effect of PTEN low on DFS within a treatment regimen was further observed in the North Central Cancer Treatment Group N9831 Intergroup clinical trial that was a Phase III, randomized, three-arm study of adjuvant trastuzumab in HER2-positive early-stage breast cancer. In this study of over 3,500 patients, 1,802 patient cases were evaluable for PTEN expression analysis by IHC. Regardless of treatment arm, one of which included patients not treated with trastuzumab, low or absence of PTEN expression did not adversely affect DFS outcome [14]; moreover, the benefit of adding trastuzumab to an adjuvant chemotherapy regimen was observed in patients regardless of PTEN status.

In another single-center study of 137 patients with HER2-positive metastatic breast cancer treated with trastuzumab, a slight trend toward shorter OS in the subgroup of patients with PIK3CA mutations was observed, yet PTEN loss was associated with a significantly shorter survival time. Consistent with the previously mentioned study that evaluated the combined effect of PTEN low and PIK3CA mutations to define PI3K pathway activation [29], PI3K pathway activation was also significantly associated with shorter OS [27].

Further evidence of the adverse prognostic effect of PIK3CA mutations in HER2-positive breast cancer was provided in a large randomized Phase III study of patients receiving docetaxel in combination with trastuzumab plus placebo or docetaxel in combination with trastuzumab plus pertuzumab. In this study, PFS was poorer in patients with PIK3CA mutation-positive disease compared with those with PIK3CA wild-type disease, independent of treatment [31].

In our study, PIK3CA mutations and PTEN low were not predictive of lapatinib efficacy as there was no significant interaction effect with each marker and treatment. However, the study is limited as it was a retrospective analysis and the number of patients with a PIK3CA mutation was small. Furthermore, the study was not powered to detect a statistically significant interaction. PTEN expression may not be predictive as both low and expression subgroups derived significant benefit with lapatinib and paclitaxel. Nevertheless, in three smaller clinical studies in patients with HER2-positive breast cancer, PIK3CA mutations and PTEN loss did not impact the efficacy of lapatinib, whether administered alone or in combination with chemotherapy [22]–[24]. A composite definition of PI3K pathway activation to include known aberrations that are proven to result in the activation of this pathway may have greater predictive effect than the individual factors alone. We analyzed the treatment effect within a subgroup that included PIK3CA mutations and PTEN low combined, and a statistically significant improvement in OS and PFS with the combination of lapatinib plus paclitaxel was observed; however, we did not directly evaluate PI3K pathway activation in this patient cohort. Our findings are in contrast to a small study (*n* = 57) of patients with HER2-positive metastatic breast cancer where PI3K pathway activation, defined as PIK3CA mutation and/or PTEN expression loss, resulted in lower CBR in patients treated with lapatinib in combination with capecitabine [13].

The inconsistencies across studies may be explained by the methodological differences used in the determination of PTEN and PIK3CA. The IHC assays used in the determination of PTEN protein expression across the breast cancer studies were not standardized; the studies reporting on the frequency of PTEN loss of expression in breast cancer varied with the use of PTEN antibody, scoring method, and definition of PTEN loss (that is, PTEN low expression versus absence of PTEN expression). Furthermore, the methods used in PIK3CA mutation analysis also varied in parameters that included sensitivity as well as which mutations were evaluated. As evident in these studies, methods that have undergone standardized validation are essential to accurately determine PTEN and PIK3CA status based on the consequential role these markers could play in aiding therapeutic decisions.

The relevance of PIK3CA mutations and PTEN low on the efficacy of HER2-directed agents has been evaluated in many patient cohorts as the presence of these alterations are implicated in resistance to trastuzumab and lapatinib [8],[9]. In our study, the efficacy results were consistent with the addition of lapatinib to paclitaxel conferring treatment benefit in patients with HER-positive metastatic breast cancer regardless of PIK3CA status and PTEN expression.

## Conclusions

Overall, PIK3CA mutations and PTEN low are not conclusively established as conferring resistance to lapatinib in patient-based studies. Prospective studies that are sufficiently powered to evaluate the interaction effect of PIK3CA mutations and/or PTEN low on HER2-directed therapies and thus provide the level of evidence required to establish their clinical relevance may be needed for future combination strategies with agents that target the PI3K pathway.

## Authors’ original submitted files for images

Below are the links to the authors’ original submitted files for images. Authors’ original file for figure 1 Authors’ original file for figure 2 Authors’ original file for figure 3 Authors’ original file for figure 4 Authors’ original file for figure 5

## Abbreviations

CBR: clinical benefit rate
CI: confidence interval
CR: complete response
DFS: disease-free survival
DNA: deoxyribonucleic acid
ECOG: European Cooperative Oncology Group
ER: estrogen receptor
FFPE: formalin-fixed, paraffin-embedded
FISH: fluorescence *in situ* hybridization
HER2: human epidermal growth factor receptor 2
HR: hazard ratio
IHC: immunohistochemistry
ITT: intent-to-treat
Lap: lapatinib
ORR: overall response rate
OS: overall survival
Pac: paclitaxel
Pbo: placebo
PD: progressive disease
PFS: progression-free survival
PgR: progesterone
PI3K: phosphatidylinositol 3-kinase
PIK3CA: PI3K catalytic subunit alpha gene
PR: partial response
PTEN: phosphatase and tensin homolog
SD: stable disease
