# Supplementary figures and images for: Association of phosphatase and tensin homolog low and phosphatidylinositol 3-kinase catalytic subunit alpha gene mutations on outcome in human epidermal growth factor receptor 2-positive metastatic breast cancer patients treated with first-line lapatinib plus paclitaxel or paclitaxel alone
Source: Breast Cancer Res. 2014 Jul 24;16:405. doi: 10.1186/s13058-014-0405-y (PMC4187249; doi:10.1186/s13058-014-0405-y)

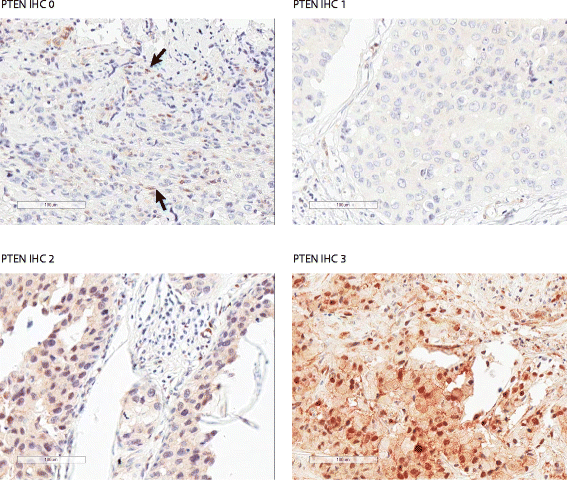

Supplement: Supplementary file 1 — Authors’ original file for figure 1 [file 13058_2014_405_MOESM1_ESM.gif]

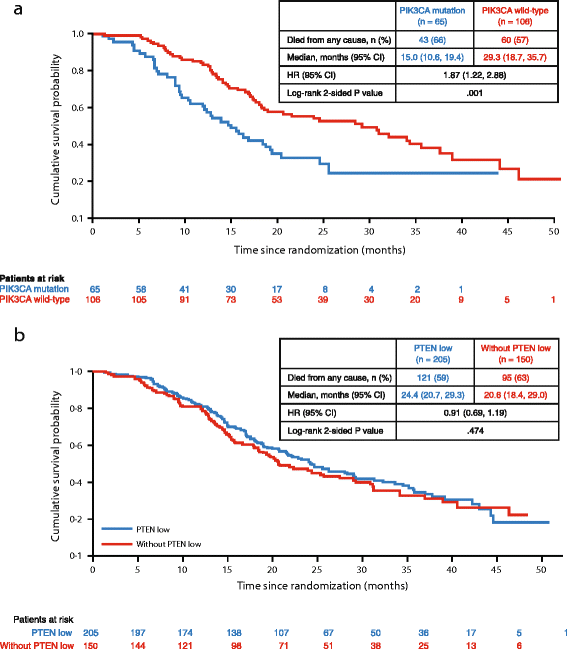

Supplement: Supplementary file 2 — Authors’ original file for figure 2 [file 13058_2014_405_MOESM2_ESM.gif]

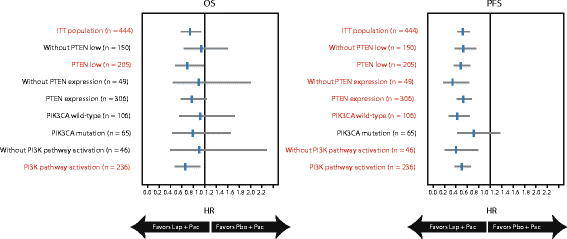

Supplement: Supplementary file 3 — Authors’ original file for figure 3 [file 13058_2014_405_MOESM3_ESM.gif]

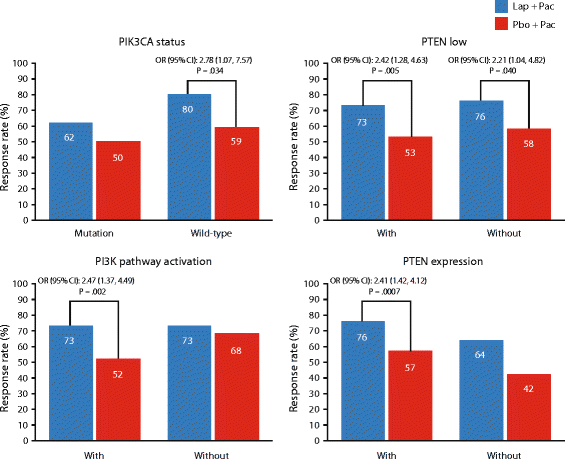

Supplement: Supplementary file 4 — Authors’ original file for figure 4 [file 13058_2014_405_MOESM4_ESM.gif]

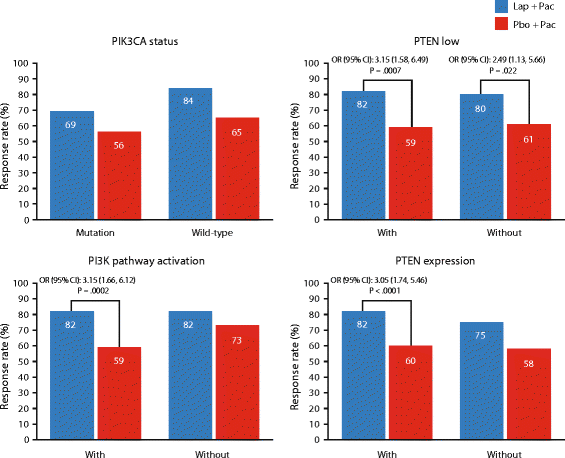

Supplement: Supplementary file 5 — Authors’ original file for figure 5 [file 13058_2014_405_MOESM5_ESM.gif]
